# Counting Cattle in UAV Images—Dealing with Clustered Animals and Animal/Background Contrast Changes

**DOI:** 10.3390/s20072126

**Published:** 2020-04-10

**Authors:** Jayme Garcia Arnal Barbedo, Luciano Vieira Koenigkan, Patrícia Menezes Santos, Andrea Roberto Bueno Ribeiro

**Affiliations:** 1 Embrapa Agricultural Informatics, Campinas 13083-886, Brazil; luciano.vieira@embrapa.br; 2 Embrapa Southeast Livestock, São Carlos 13560-970, Brazil; patricia.santos@embrapa.br; 3 Universidade Santo Amaro, UNISA, UNIP, São Paulo 04743-030, Brazil; arbribeiro@prof.unisa.br

**Keywords:** unmanned aerial vehicles, Canchim breed, Nelore breed, convolutional neural networks, mathematical morphology

## Abstract

The management of livestock in extensive production systems may be challenging, especially in large areas. Using Unmanned Aerial Vehicles (UAVs) to collect images from the area of interest is quickly becoming a viable alternative, but suitable algorithms for extraction of relevant information from the images are still rare. This article proposes a method for counting cattle which combines a deep learning model for rough animal location, color space manipulation to increase contrast between animals and background, mathematical morphology to isolate the animals and infer the number of individuals in clustered groups, and image matching to take into account image overlap. Using Nelore and Canchim breeds as a case study, the proposed approach yields accuracies over 90% under a wide variety of conditions and backgrounds.

## 1. Introduction

The management of large livestock areas adopting extensive production is challenging and, in many cases, deficient [1]. With the popularization of Unmanned Aerial Vehicles (UAV, also known as Unmanned Aerial Systems or drones), a high-resolution, low cost bird’s eye view of the property can be acquired. As promising as this technology is, the realization of its full potential is hindered by the fact that the extraction of relevant information from images captured using the UAV is far from trivial. In the case of livestock population estimation, difficulties arise from animal movement, landscape variety (bare soil, dry and vigorous pasture, etc.), occlusions by obstacles like trees and sheds, and the tendency of animals to group together.

The use of UAVs for cattle monitoring is still in its infancy. Some applications do not explore the imaging capabilities of the UAV, rather using the aircraft for actions such as cattle round up [2] and collection of data from sensors fitted on the animals through wireless communication [3,4,5]. Other studies used the images for direct visual analysis aiming at cattle detection [6], measurement of the distance between cow and calf [7], and determination of feeding behavior [8]. In most cases, however, images captured by UAVs are used to feed an algorithm for extraction of relevant information.

With the exception of the work by Longmore et al. [9], in which thermal images and an astronomical source detection software were used for detection of cattle, all studies for detecting and counting cattle use some kind of deep learning technique. Three different approaches are usually employed to perform the task. The first approach, semantic segmentation, associates each pixel in the image to a class label. Although there are several deep learning architectures capable of performing semantic segmentation (e.g., U-Net [10], DeepLab [11] and FastFCN [12]), this kind of technique has not yet been applied to cattle recognition and counting, probably due to the difficulty involved in the annotation of the reference data [13]. The second approach, object detection, delineates a box bounding the objects of interest. One of the most used object detection technique is YOLO [14], and its second version was used to detect cattle in a few studies [15,16]. Another algorithm, R-CNN, was used in Reference [17] for counting sheep. The third approach uses regular Convolutional Neural Networks (CNN) architectures to generate heat maps (probability distributions) that theoretically pinpoint the position of the animals in the image [18,19,20].

Each of the three approaches mentioned above have strengths, but also a few weaknesses. Semantic segmentation requires large amounts of data to be annotated at pixel level, in a process that can be very labor intensive and time consuming. In addition, the non-cattle class has very high variability, thus requiring a large number of samples to be properly characterized in the reference dataset. Object detection techniques usually have some conditions under which they perform poorly. Since animal configurations (body size and orientation, degree of clustering, etc.), in the field vary considerably, errors may be common. The extraction of probability distributions using CNNs often leads to issues when dealing with crowded images, especially if the animals are grouped together [20]. In addition, as discussed by Barbedo et al. [13], although the results reported in the studies mentioned above are encouraging, the experimental designs have some limitations that make it difficult to evaluate the real significance of the reported results.

This article proposes a four-step approach combining the inference capabilities of deep learning models with finely tuned image processing for extraction of relevant information. In the first step, which was described in detail in a previous work [13], each image is divided into squares using a regular grid, and squares containing parts of animals are identified by means of a CNN. In the second step, color space manipulations are used to enhance contrast between animals and background and thresholds are applied to generate binary masks. In the third step, the masks are combined using binary operations, and morphological operations are used to separate clustered animals. In the fourth step, images are matched using feature matching, providing the information needed to tally the final count estimate.

The method proposed in this article shares some similarities with the third approach described above (CNN-based probability distributions), as both use deep learning models to classify regions according to the presence or absence of animals. The main difference of the strategy adopted in this work is that it includes a step specifically designed to deal with clustered animals, which is often the main source of error in other approaches [20]. It is also worth noting that the image dataset used in the experiments includes a wide variety of conditions, with images being captured under different weather conditions (sunny and overcast), at different times of the day and of the year, and with different pasture conditions. Such a variety aimed at guaranteeing that the results are as realistic as possible.

In summary, the main contribution of this work is that the proposed algorithm is capable of providing accurate estimates for the number of animals even under difficult conditions such as: (1) presence of clustered animals that are difficult to separate even using sophisticated deep learning models; (2) changes in the contrast between animals and background, which is common due to the heterogeneity of cattle farms; and (3) illumination variations, which are common under real natural conditions. The algorithm can also handle animal movement, although there is room for improvement.

It is worth noting that although the CNN models used in the algorithm successfully deal with poor contrast and illumination [13], they only provide a rough estimation for the position of the animals. Determining how many animals are actually present in the selected regions is challenging under these conditions due to difficulties in animal delineation. For this reason, the proposed algorithm has many steps specifically designed to deal with each problematic situation. It is also important to highlight that the CNN models described in Reference [13] were employed without modification, that is, there were no new experiments regarding the deep learning part of the algorithm.

## 2. Materials and Methods

### 2.1. Image Collection

Two different image datasets were used in this investigation. The first one, which is the same used in Reference [13], was captured using a DJI Phantom 4 Pro equipped with a 20-MPixel camera. The second one was built using a DJI Mavic 2 Pro, also equipped with a 20-MPixel camera. Missions were carried out at the Canchim farm, São Carlos, Brazil (21°58′28″ S, 47°50′59″ W) at 11 dates over the year of 2018 (DJI Phantom 4 Pro) and 1 date in 2019 (DJI Mavic 2 Pro). Camera settings were all kept on automatic, except exposition, which used the presets “sunny” and “overcast” depending on weather conditions. Images were saved in the 8-bit JPEG format. In all cases, images were captured from an altitude of 30 m with respect to the take-off position. This altitude provided a fine GSD (approximately 1 cm/pixel) without disturbing the animals, which showed no reaction when the aircraft flew over them. Altitude and GSD had variations of up to 20% due to the ruggedness of the terrain. Frontal and side image overlap were both set to 70%. Each animal is represented by 13,000 pixels in average—this number varies considerably with the size and position of the animals, as well as with the actual altitude at the moment of capture. The images cover a wide range of capture conditions (see details in Reference [13]), and animals from both Canchim and Nelore breeds were present during flights. The first dataset contained 19,097 images, from which 1853 images having at least part of an animal were selected for the experiments. The second dataset contained 826 images, from which 118 images containing animals were selected for the experiments. The main difference between the datasets is that the second includes several images containing more than 100 animals, while in the first dataset no image contains more than 16 individuals. In addition, the second dataset includes numerous calves, which further complicates the detection process.

### 2.2. Cattle Detection Using Deep Learning

The detection of cattle using a regular grid and deep learning was thoroughly explored in Reference [13], so no further experiments in this regard were carried out in the context of this work. NasNet Large [21], which consistently provided accuracies above 98%, was chosen as the trained CNN model responsible for classification. Images are divided into 224 × 224 pixel squares by a regular grid, and each individual region is labelled as “cattle” or “non-cattle”. Non-cattle regions are discarded and only the remaining cattle regions are used in the experiments described in the following. With the exclusion of regions without animals, the detection of spurious objects in the next steps of the algorithm was greatly reduced.

### 2.3. Study Design

The algorithm described in the remainder of this section was optimized using 10% of the complete dataset (197 images). Different rules and parameter values were tested exhaustively under a wide variety of conditions in order to determine the combination that would be the most robust to a wide range of circumstances. It is worth noting that many of the rules described in this section are only valid for the breeds considered in this study.

### 2.4. Color Space Manipulation

**Figure 1:**
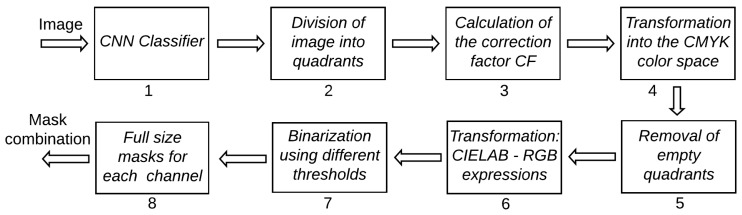
Steps adopted to generate the binary masks used for animal segmentation.

**Figure 2:**
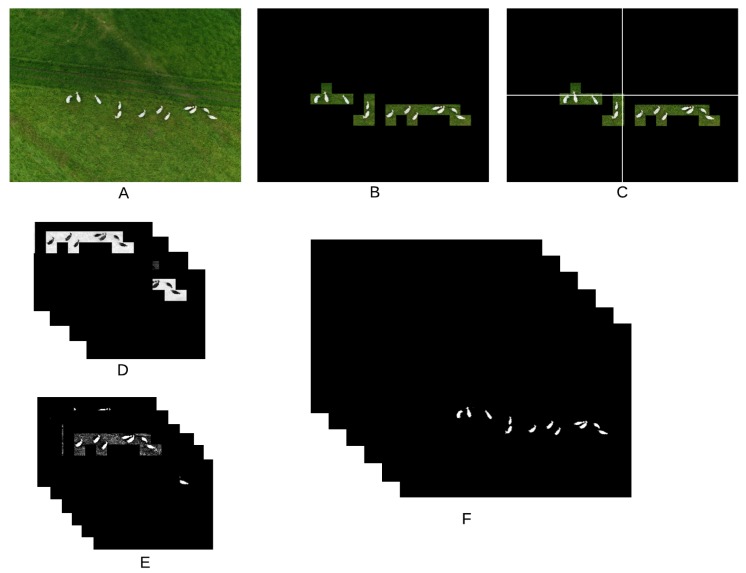
Examples of images resulting from each processing step shown in Figure 1. (**A**) Original image; (**B**) region of interest; (**C**) quadrants of the image; (**D**) color channels used in the algorithm; (**E**) masks associated to each channel; (**F**) six resulting masks with all quadrants united.

The color of the animals in the images ranged from white to light beige, with some darker coating occurring in some animals. Background colors were much more varied—depending on the time of the year, pasture color ranged from bright green to pale yellow; bare soil color ranged from reddish brown to light yellow. Due to the variation in contrast, separating animals from the background proved to be a difficult task that required the use of a complex set of rules. Figure 1 shows all steps adopted for color manipulation, and Figure 2 shows examples of the images generated after each step. Each step is explained in detail in the following, using the numbers associated to each box in Figure 1 as reference.

After the region of interest containing the animals is determined by means of the CNN (1), the image is divided into four quadrants (2). Each quadrant is processed separately in order to take into consideration possible illumination differences inside the image. A correction factor (cf) is calculated for each quadrant according to the expression $cf=85/mean(P_{i})$, where $P_{i}$ are the values of all pixels in all three color channels (3). Images are transformed from the RGB to the CMYK color space (4), from which only the channel K is not taken for further processing. The cyan channel C is useful to detect darker animals in dark images; the magenta channel M is effective in highlighting shadowed parts of the animals while avoiding problems with bare soil; the yellow channel Y is the best for detection of animals under normal conditions. If the number of pixels in the channel Y with value larger than zero is smaller than 50,000, the quadrant has too little useful content and is discarded (5). Another color transformation is applied to the original images using the mathematical expressions used to transform from the CIELAB to the RGB color space (6), but in this case those expressions are applied to the RGB channels as if they were the L, a and b channels of the CIELAB color space. This transformation has no meaning perceptually, but it was very effective highlighting darker parts of the animals. Only the third channel (custom color channel) of this transformation is taken into consideration for further processing. In all four color channels considered in this work, animals appear darker than their surroundings, meaning that, in the generation of the binary masks, pixels below the calculated threshold value are always made white (7). The thresholds are then used to generate the binary masks for each quadrant. The threshold for the C channel is given by min(100,40·cf), where cf is the correction factor calculated for each quadrant, as described before. The threshold for the M channel was simply set to 30, as this value was generally successful in generating useful masks. In most cases, channel Y was the one that provided the best contrast between animals and background. However, it was also the one that required the most complex set of rules for the threshold calculation. First, a 256-point histogram is generated for each quadrant, and a moving-average filter using a sliding window of length 25 is applied to smooth the histogram curve, thus removing spurious peaks and valleys. If the sum of the first 10 points of the histogram is larger than the sum of the last 156 points, the quadrant is deemed very dark and the threshold is set to 10. If the quadrant is not too dark, the peaks of the histogram are located. When peaks are separated by less than 30 points, only the larger one is kept for further processing. At this point, it is expected that at least two peaks remain (one related to the background and one to the animals). If only one peak remains, the threshold for the quadrant is discarded; otherwise, only the first two peaks are kept. Then, all points between both peaks with amplitude smaller than 10% of the second peak are located. The index of the point closest to the second peak that satisfies this condition is taken as the quadrant threshold. If no point satisfies the 10% condition, the threshold is set as the index of the point with smallest amplitude between the peaks. Then, a global threshold, given by the smallest threshold among all quadrants, is applied to the whole image (instead of applying different thresholds to each quadrant). It was observed that the conditions under which the image is captured may cause large changes in the way the Y channel behaves. Because of that, this global threshold is applied twice more after being divided by 2 and 4, thus generating three separate masks for the Y channel. Finally, a threshold given by min(150,120·cf) is used to generate the mask for the custom channel. The six binary masks resulting from the proper reintegration of all quadrants (8) are the inputs for the next stage of the algorithm.

### 2.5. Mask Combination

**Figure 3:**
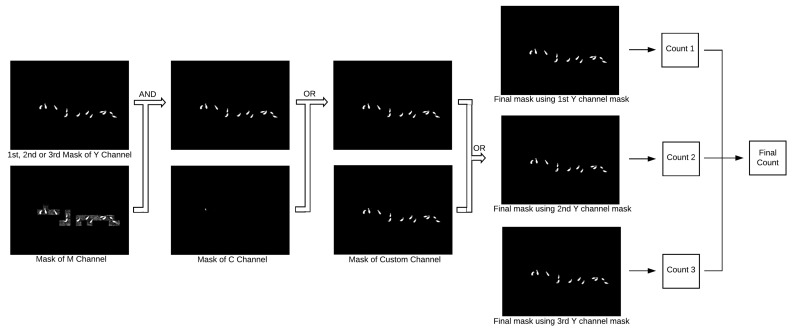
Mask combination and estimation of the number of animals.

Figure 3 shows how the different masks are combined together. First, the masks for the Y and M channels are combined using the AND operation, that is, pixels in the resulting mask will be white if their counterparts in both original masks are also white. If the original objects in the Y mask lose area after the AND operation but are not completely removed, the blackened pixels are reverted back to white. The resulting mask is merged with the C mask by an OR operation, that is, the resulting pixels will be white if any of their counterparts in the original masks are also white; finally, the mask from the second color transformation is incorporated also by applying the OR operation.

### 2.6. Estimation of the Number of Animals

As indicated in Figure 3, three estimates for the number of animals are calculated, each using the final mask obtained using a different threshold for the Y channel. Several rules are chained to produce each estimate, as described below:

(a) All objects in the resulting mask are identified, and the area and solidity (ratio between the actual area and the area of the smallest convex polygon encompassing the object) are calculated for each of them.
(b) Objects with area smaller than 2000 are removed; the same is done with objects with area smaller than 5000 and solidity smaller than 0.7.
(c) A preliminary estimate for the number of animals is given by the number of connected objects in the image.
(d) A new correction factor (ev) is calculated to take into account differences in the GSD due to elevation variations; ev=1 if the mean area of the objects ($A_{o}$) in the image is smaller than 8000, 1.25 if $8000\leq A_{o}<15,000$, and 1.5 if $A_{o}\geq 15,000$.
(e) Objects are considered as potentially containing more than one animal if their area is larger than ev·15,000, their solidity is smaller than 0.65, or their area is larger than ev·12,000 and solidity is smaller than 0.7.
(f) For each object identified in the previous item, two morphological operations are applied. First, an 20ev-pixel thinning is applied, followed by a 1-pixel erosion. The area and solidity of the resulting fragments are then calculated. The number of animals associated to each object is given by $N=A_{h}+S_{l}-1$, where $A_{h}$ is the number of fragments with area larger than 100 and $S_{l}$ is the number of fragments with solidity smaller than 0.5.
(g) Results are compiled for all objects, providing an estimate for the number of animals in the image.

The final estimate (Final Count in Figure 3) is given by the highest estimate, provided that such a number is not more than three units larger than the second highest estimate, in which case the latter is taken as the final estimate.

The proposed algorithm employs several tunable parameters that were set specifically for the conditions found in this study. Table 1 shows all tunable parameters, the values adopted in this work, and how they should be adjusted in case new conditions are considered.

### 2.7. Image Matching and Final Population Estimate

All images captured in the field are georeferenced; however, due to GPS imprecisions, matching images based only on the recorded coordinates is not appropriate. There are several well established methods for feature extraction and feature matching [22]. SURF (Speeded Up Robust Features) [23] was used as the primary feature extractor, with BRISK (Binary Robust Invariant Scalable Keypoints) [24] being used when the number of matched points using SURF was smaller than 6. It is worth noting that the method used for image matching is not important, as long as it yields accurate results. Because neighbor images had an overlap of at least 70%, their matching was successful in all cases.

As images overlap, the same animal will appear in multiple images. In order to avoid overestimations, each animal should be considered only once. Initially, the idea was to track each animal from image to image. When animals are isolated, this task is relatively simple, but when they appear in groups (which is very common), this is quite difficult to be achieved. The main difficulty resides in the fact that animals move, changing positions among themselves and also altering their orientation with respect to the camera. This makes animal tracking very challenging, and more research is needed in this regard. Instead, the following algorithm was applied:

- Images containing animals are analyzed sequentially according to their chronological order.
- Objects (animals or clusters of animals) located at the borders of the image are identified. Those animals/clusters are removed from the original number estimation, and their positions are stored to be used later.
- Animals located in the regions that overlap with images already analyzed are also removed, except in the case of those that were located at the borders of previous images.
- After all images have been analyzed the results are tallied, revealing the final count estimate.

### 2.8. Experimental Setup

As mentioned before, the part of the algorithm using deep learning to define the region of interest was thoroughly investigated in [13], so no further experiments in this regard were carried out.

Results were tallied at three different levels: objects, individual images and entire areas.

As mentioned before, objects are defined as individual animals or clusters containing multiple individuals. In order to test the hypothesis that larger clusters are more prone to errors, objects were classified according to the number of animals present in the cluster, and a confusion matrix crossing actual and estimated counts for each of these classes was generated. Precision, recall and F1-scores were also calculated for each cluster size. Results considering only calves were also computed.

At the image level, a few measurements were computed besides precision, recall and F1-scores. The mean deviation is given by $(\sum \left(C_{e}\left(n\right)-C_{t}\left(n\right)\right))/N$, where $C_{e}$ and $C_{t}$ are the estimated and actual number of animals in each image *n*, and *N* is the total number of images. The standard deviation of the error is given by $\sqrt{\sum |C_{e}\left(n\right)-C_{t}\left(n\right)|}/N$. The largest positive and negative deviations from the actual value were also computed. In order to better understand the sources of error, precision, recall and F1-scores were also calculated considering only images containing trees, white sheds and feeders, as all these structures had the potential to be mistakenly detected as animals.

While the results for individual objects and images were computed before image matching, the whole algorithm was applied when the entire area of interest was considered. Here, estimated and actual counts were directly tallied for the areas imaged in each of the 12 dates in which flight missions were carried out.

## 3. Results

**Figure 4:**
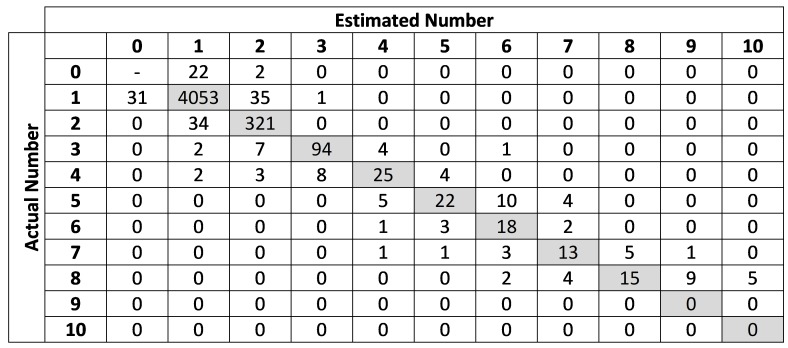
Confusion matrix crossing actual and estimated counts for different cluster sizes.

Figure 4 shows how the algorithm performed considering different sizes of clusters. Because the number of samples for each cluster size is very different, the matrix shows the absolute values instead of percentages. Precision, recall and F1-scores for each cluster size are presented in Table 2.

Due to the adoption of rules based on the typical size of animals, there were some problems detecting calves when they were touching other animals: in isolation, 99% of the calves were detected, while only 71% of those were properly accounted for when in clusters.

Table 3 shows the results obtained at the image level. The results were obtained considering all individual images in dataset, except those used for optimization of the algorithm.

Table 4 shows actual and estimated animal counts for each of the 12 dates in which flight missions were carried out.

## 4. Discussion

As expected, the proposed algorithm was nearly perfect detecting animals in isolation (Figure 4). The few errors in this case were due to the morphological operations—some animals were completely removed by the thinning and erosion operations, thus remaining undetected, while others had their bodies broken into two or more objects, leading to multiple detections. Error rates tended to be higher as the number of clustered animals increased. However, it is worth noting that even in those cases the algorithm yielded reasonably good estimates, with errors rarely exceeding ±1. Size rules worked well even though differences in GSD, animal size and animal position were significant. There was also a balance between under- and overestimations, a fact that is reflected by precision and recall having exactly the same values over the entire dataset (Table 2). This indicates that the algorithm was tuned properly to avoid bias.

**Figure 5:**
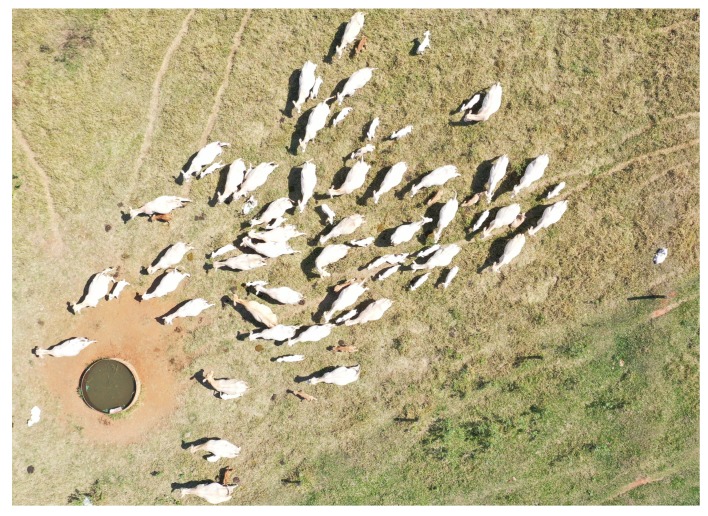
Example of image containing many calves.

The results were similar when whole images were considered (Table 3). The highest error observed was −20, which was associated with an image containing several calves (Figure 5), which tend to remain undetected when touching other animals due to their comparatively small size. In all other cases, the error never surpassed ±3, and the overall mean error was exactly zero, again confirming that the algorithm is well balanced. It is worth noting that the calves in the images were very young, which means they spent considerable time nursing; as calves get older, they tend to spend less time attached to their mothers.

**Figure 6:**
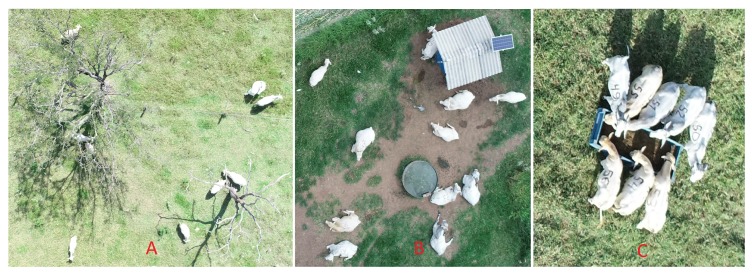
Examples of images containing trees (**A**), shed (**B**) and feeder (**C**).

Not many trees were presented in the dataset, and many of those were defoliated (Figure 6A), so animals were at least partially visible at all times. As a result, they had little impact on the overall results. Areas with significant tree coverage may require some kind of statistical correction to account for occluded animals, but this was not tested here due to the lack of images containing significant tree cover. Several images contained sheds with white roofs. While the CNN model was successful in removing those from the region of interest, at least part of those structures remained when there were animals nearby (Figure 6B). This almost invariably led to overestimation of the number of animals. Feeders with a light shade of blue were also common, and as in the case of sheds, they often led to overestimation, especially when there were several animals feeding together (Figure 6C). Many cattle farms do not adopt supplementary feeding, in which case no feeders will be present.

In the case of whole image areas, the only relatively large deviation from the actual number of animals was observed for the second dataset (last date in Table 4), mostly due to the presence of several calves. For the first dataset, the count estimates obtained for the whole areas tended to be slightly underestimated. This slight imbalance was due to specific animal movement patterns observed when flights took place, and not to an inherent algorithm bias. The strategy adopted to deal with image overlap is susceptible to errors due to significant animal movement. Ideally, each animal should be tracked across several images, but as discussed before, this is still difficult to achieve. If animal movement could be considered random in a certain area, the two types of error (missed animals and multiple counts of the same animal) would tend to cancel out. However, since there are several factors that influence animal behavior [25], this assumption may not hold in most cases. One way to minimize this problem is to capture the images when animals tend to move less. Studies dedicated to cattle behavior have determined that animals rarely spend more than 1 h/day walking, thus much of the movement activity is due to grazing [25]. Grazing activities are more intense close to sunrise and sunset, and if the green forage mass is high, animals will not need to move much to feed properly [25]. In general, flying in the period between 1 h after sunrise and 1 h before sunset seems to be appropriate. It is also worth noting that animals tend to seek shelter under trees and other structures when temperatures are uncomfortably high, so midday flights should be avoided in hot days. Depending on the angle of insolation, shadows may be very pronounced, but the color scheme adopted in the algorithm is able to compensate most of potential adverse effects.

As mentioned before, images were captured under a wide variety of conditions. Animals and background had a wide range of colors associated, which caused the level and type of contrast between them to vary significantly. Although color manipulations and carefully crafted rules were mostly successful in separating animals from background, there were a few cases in which errors occurred. Two situations, in particular, led to difficulties. First, due to specular reflections, some patches of bare soil had a light coloration, mimicking the typical shades expected for the animals. Second, certain animals had a relatively dark shade of yellow which had very little contrast with most areas of bare soil and dry pasture. A degree of confusion between animals and background will almost always be present, regardless of the breed and characteristics of the pasture, unless the experimental conditions are tightly controlled. Fortunately, in this study there were very few instances in which the algorithm was not able to handle these difficult situations, at least partially.

**Figure 7:**
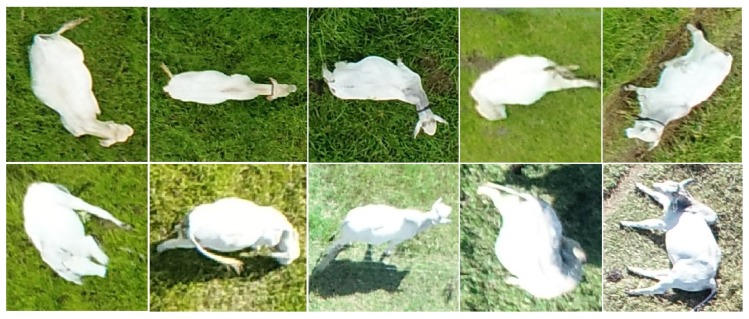
Examples of animals in different positions.

The positions of the animals also varied significantly (Figure 7). This not only makes it difficult to track animals, as mentioned before, but it also causes object-size-based rules to fail due to variations in the areas of the animals that are visible in each instance. Position variation has little impact when the animal is isolated, but causes difficulties in clusters. This was in fact the main cause of the misestimates shown in Figure 4, and a suitable solution for this problem will be the object of future investigations.

**Figure 8:**
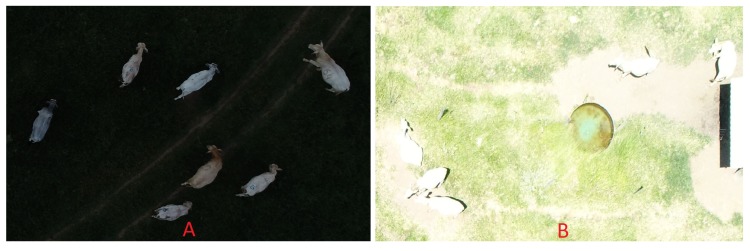
Examples of images with underexposure (**A**) and overexposure (**B**).

Problems with the automatic camera setup and with the mission planning caused a few images to be too dark or too bright due to incorrect exposure, which in turn was caused by illumination variations in partially cloudy days. Although this kind of problem can be easily avoided in practice, it seemed useful to investigate how the algorithm would deal with poor capture conditions. In the case of dark images, the range of pixel values became limited to the lower portion of the scale (Figure 8A). In other words, there is less information available to properly detect the objects of interest in the image. In most cases, even this limited range of pixel values is enough for proper animal counting. On the other hand, in cases with limited contrast between animals and background, this loss of information may lead to error, which indeed was the case in the few instances where this occurred. The same was observed for excessively bright images, with one additional problem. Depending on the angle of insolation, specular reflections (excessive glare) were quite noticeable, but mostly manageable. However, in combination with excessive exposure, specular reflections caused animals and background to become nearly indistinguishable (Figure 8B), inevitably leading to error. Incorrect exposure can be easily avoided in practice, but the images produced under these circumstances were useful to determine how the algorithm would handle extreme conditions. It was observed that, except in the cases with extreme loss of information, the algorithm was able to deal with these abnormal circumstances.

The main shortcoming of the proposed algorithm is that many parameters need to be set by hand whenever images are expected to be captured under new conditions (different resolution, different cattle breeds, pastures with different characteristics, etc.). Deep learning techniques for object detection and localization [14] can potentially be more robust to condition changes, but the amount of images needed for training a model capable of dealing with most possible situations may be prohibitively large. Given the limitations of current datasets, retraining of the models will be needed whenever new conditions are to be considered. This stresses the importance of data sharing for more general solutions to be feasible, no matter the approach being adopted.

## 5. Conclusions

Counting cattle using UAVs is a deceptively hard problem which has associated many factors that may impact the way animals are detected and counted. To deal with such a difficult problem, the proposed method chains four distinct modules: CNN to delineate the region of interest, color transformation and manipulation to segment animals from background, mathematical morphology to separate clusters and eliminate spurious objects, and image matching to compensate for image overlap. Chaining several modules carries the risk of errors from each module propagating to the next, derailing the whole estimate. However, the experiments revealed that the proposed structure is accurate and robust to different types of situations.

Some situations have been shown to pose a challenge, especially the lack of contrast between animals and background, animal movement, large animal clusters and the presence of calves. There are a few potential solutions expected to be investigated in the near future. Semantic segmentation may replace the two first modules of the algorithm and avoid the need for handcrafted color rules, and new animal tracking approaches based on more subtle cues will also be tested. The possibility of covering large areas at once by capturing the images at an oblique angle, thus removing the need for long flights and image overlaps, is also under investigation.

Those challenges, although difficult to overcome, had a relatively mild impact on the overall accuracy of the proposed algorithm. Under normal population densities of up to four animals per hectare [26], the algorithm is expected to produce accurate estimates. Higher population densities can also be handled adequately, as long as the number of young calves is not too high. There is, however, room for improvement. The strategy used to account for multiple appearances by the same animal is not optimal; new solutions for animal tracking will be investigated in future experiments. Future work should also concentrate on new ways to capture images, having as main goal to increase the usable area available in each image (for example, by capturing images at an oblique angle). Efforts should also be directed toward capturing images for other cattle breeds, making it possible to extend the algorithm’s applicability. The effects of GSD variations on the ideal parameter values is also a topic of interest for future investigations.

Although the algorithm described in this article was developed having cattle counting in mind, the methodology can be adapted to some other applications such as ship detection [27], tent detection in refugee camps [28], among others. 

## Figures and Tables

**Table 1:** Tunable parameters used in this study.

Parameter	Value Adopted	Adaptation to Different Conditions
*cf*	85/mean(Pi)	Should work under any condition
Minimum number of active pixels in a quadrant	50,000	Should work under any condition
Threshold for the C channel	min(100,40·cf)	This parameter was set to detect darker animals under poorly lit conditions; it may be discarded if such conditions are not present
Threshold for the M channel	30	This parameter was included to address the problem of redish soil being identified as animals; it may be discarded if such conditions are not present
Threshold for the Y channel	Variable	This channel showed the best discriminative potential for light animals; with darker animals, the C channel may be more suitable as the main source
Threshold for the custom channel	min(150,120·cf)	This channel was used to resolve animals when contrast with the background was poor; this parameter may change when breeds with different coat colors
Size rules	Varied	All size rules were derived considering a GSD of 1 cm/pixel; the adopted values should be rescaled according to the GSD adopted in each case
Solidity rules	Varied	Should work under any condition

**Table 2:** Precision, recall and F1-scores for each cluster size.

Cluster Size	Precision	Recall	F1-Score
1	99.1	99.2	99.1
2	100.0	95.0	97.4
3	97.6	96.2	96.9
4	96.2	83.3	89.3
5	85.9	95.7	90.5
6	98.2	95.6	96.9
7	92.9	91.9	92.4
8	86.3	93.8	89.9
All	97.4	97.4	97.4

**Table 3:** Results for images containing different types of structures.

Structure	Precision	Recall	F1-Score	Mean Dev.	Std. Dev.	Min. Dev.	Max. Dev
All	97.4	97.4	97.4	0	0.73	−20	+3
Trees	97.4	93.4	95.4	−0.42	0.80	−2	+1
Sheds	87.1	98.4	92.4	+1.11	1.32	−1	+3
Feeders	94.9	96.7	95.8	+0.49	0.99	−2	+2

**Table 4:** Count estimates for the whole areas imaged.

Date	Estimated	Actual
January 9, 2018	86	88
February 9, 2018	105	112
March 14, 2018	80	83
April 16, 2018	74	78
April 17, 2018	144	142
May 17, 2018	43	43
June 18, 2018	29	29
June 22, 2018	43	44
July 20, 2018	31	30
August 21, 2018	22	22
November 29, 2018	79	76
September 9, 2019	199	242
Total	935	989
